# Supplementary material for: Prevalence and risk factors of hypertension among clients seeking care at Selected Healthcare Facilities in Kenya
Source: PLoS One. 2025 Oct 29;20(10):e0334255. doi: 10.1371/journal.pone.0334255 (PMC12571272; doi:10.1371/journal.pone.0334255)
Supplement: Attachment — Submitted filename: Hypertension - 20th Feb 2025.docx [file pone.0334255.s002.docx]

# **Prevalence, Demographic and Associations of Patients with Hypertension Seeking Care at Different Healthcare Facilities in Kenya**

# **Authors:**

**Jasmit S Shah**, PhD, Aga Khan University, Nairobi
**Soraiya Manji**, MMed, Aga Khan University, Nairobi
**Cynthia Smith**, MSc, Aga Khan University, Nairobi
**Jamila Nambafu**, MMed, PCEA Chogoria Hospital
**Anthony Ochola**, MMed, Tenwek Hospital  **Linda Barasa**, MMed, Avenue Hospital
**Faraj Amir**, MbChb, Garissa County Referral Hospital
**Husni Abdalla**, MMed, Aga Khan Hospital, Mombasa
**Simeon Jowi**, MMed, Kericho County Referral Hospital
**Caroline Mithi**, MMed, Kenyatta University Teaching and Referral Hospital

**Rajiv Patel,** MMed, Aga Khan University, Nairobi
**Sayed K Ali**, MD, Aga Khan University, Nairobi

**Corresponding Author:**
Sayed K Ali
[sayed.karar@aku.edu](mailto:sayed.karar@aku.edu)
+254 721260557

Jasmit S Shah

[jasmit.shah@aku.edu](mailto:jasmit.shah@aku.edu)

+254 736032935

Word Count: 7458

# **ABSTRACT**

**Background:** Hypertension remains one of the primary risk factors for cardiovascular disease and the leading cause of mortality worldwide. According to the World Health Organization (WHO), as estimated 1.28 billion adults suffer from hypertension worldwide and approximately half are unaware of the problem.. This study aimed to explore the prevalence of hypertension, and the association of sociodemographic, behavioral, and physiological factors related to hypertension in patients seeking care at the different healthcare facilities in Kenya.

**Methodology:** We carried out a cross-sectional survey study between April 2023 and July 2023. The general adult public visiting the outpatient clinics were recruited from 8 healthcare facilities in Kenya.

**Results:** A total of 1444 patients were recruited and included in the analysis. The median age of participants was 47.0 years, 54.3% were females, 75.1% were married, and 42.9% reported living in rural areas. The prevalence of hypertension was found in 29.4% of patients of which 48.5% lacked awareness of their diagnosis. Of the patients who knew of their diagnosis, 53.1% were not at goal blood pressure. Of note, 39.1% of participants with hypertension were from faith-based institutions usually serving rural areas, 31.5% from public institution and 29.4% from private institutions (p<0.001). Type of facility, age, gender, education, marital status, body mass index and residence were associated with hypertension (p<0.05).

**Conclusion:** The study highlights a significant burden of hypertension among adults attending outpatient clinics in Kenya. Nearly half of hypertensive individuals were unaware of their condition, and among those diagnosed, more than half did not achieve target blood pressure levels, indicating gaps in screening, awareness, and management. These findings emphasize the need for targeted interventions, including improved screening, awareness campaigns, and enhanced treatment strategies to improve hypertension control in diverse healthcare settings.

# **INTRODUCTION**

Hypertension remains the most important risk factor for cardiovascular disease and the leading cause of mortality worldwide. According to the World Health Organization (WHO), an estimated 1.28 billion adults suffer from hypertension worldwide causing approximately 7.6 million premature deaths [1]. Approximately half of all adults with hypertension are unaware of their condition. Furthermore, only about 1 in 5 individuals with hypertension have their blood pressure under control. There has been a shift in the burden of hypertension, with 66% of patients with hypertension living in low- and middle-income countries (LMICs) [1, 2]. The Pan-African Society of Cardiology has identified hypertension as the top priority of action to help curb and reduce the incidence of heart disease and stroke on the African continent. However, very few countries in sub-Saharan Africa (SSA) have developed and implemented clear hypertension policies related to diagnosis and management of hypertension [3].

In Kenya, among adults aged 18-69 years, approximately 25% are estimated to be hypertensive whereas 50% are estimated to be pre-hypertensive [2, 4, 5]. The prevalence of hypertension in Kenya and SSA has been steadily rising, contributing to an increased burden of cardiovascular diseases (CVD) [6]. Notably, only about a quarter of the affected patients are aware of their diagnosis, only about 18% receive treatment, and about 7% achieve hypertension (blood pressure) control [6]. Despite the strong evidence demonstrating that hypertension treatment reduces the incidence of CVD and lowers the mortality rates, this has not been effectively implemented in practice among SSA [6]. A national cross-sectional study conducted by Pengpid and Peltzer in Kenya, showed that from their study cohort, 28.6% had hypertension, and among the hypertensives, only 29.4% were aware, only 6.5% were currently using antihypertensive medication and a mere 12.5% had blood pressures less than 140/90 mmHg [7]. Similarly, Mohammed et al, in their study looking at hypertension and their determinants in Kenya, found 24.5% had hypertension (age standardized) and only 15.6% were aware of their diagnosis [8]. Of the population aware of their hypertension, only 27% were on treatment and approximately half had achieved blood pressure control [8]. Another study looking at the prevalence of hypertension in slum dwellers in Nairobi, also found that the prevalence of hypertension in this specific population was about 12% with about 20% being aware of their diagnosis, 47% were on some sort of treatment and 27% had blood pressures less than 140/90 mmHg [9].

Okello et al, conducted a study examining the prevalence, awareness, treatment, and control of hypertension and predicted 10-year cardiovascular disease risk across seven communities in Kenya, Nigeria, Tanzania and Uganda [10]. The mean age of the participants was 39.7 years, with 25.4% reported to have hypertension and about half of whom were aware of their diagnosis, 50.5% of those diagnosed were taking medication, and only 47.3% of those on medication had controlled blood pressure [10]. These findings suggest substantial room for improvement in hypertension diagnosis and treatment.

Hypertension, the leading single cause of morbidity and mortality worldwide, is a growing public health problem in SSA. With very few studies that estimate and compare the burden of hypertension across different SSA populations, more studies need to be conducted clinically to truly understand this growing disease. From the few studies that have been published; factors that have been found to be associated with hypertension are population group, older age, higher body mass index (BMI), diabetes, lower and higher level of education, family history, and alcohol and tobacco use. Caffeine abuse has also been noted to have a negative effect on the prevalence of hypertension [11]. The prevalence of hypertension and pre-hypertension are high and differ by the population group defined by occupation and degree of urbanization. In Kenya, 1 out of 10 women suffer from hypertension [12]. Only half of the populations with hypertension are aware of their diagnosis, indicating that there is a high burden of undiagnosed and uncontrolled high blood pressure in our population as SSA [12].

This study aims to explore the prevalence of hypertension, and the association of sociodemographic, behavioral, and physiological factors related to hypertension in clients seeking care at the different healthcare facilities (including public, private, and faith-based) in Kenya.

# **MATERIAL AND METHODS**

We carried out a cross-sectional survey study between 1^st^ April 2023 and 31^st^ July 2023. The general adult public visiting the outpatient clinics were recruited from eight different healthcare facilities: Aga Khan University Hospital, Nairobi; Aga Khan Hospital, Mombasa; Avenue Hospital, Nairobi; Garissa County Referral Hospital, Garissa; Kericho County Referral Hospital, Kericho; Kenyatta University Teaching and Referral Hospital, Nairobi; PCEA Chogoria Mission Hospital; Tharaka-Nithi and Tenwek Hospital, Bomet. Aga Khan University Hospital, Aga Khan Hospital and Avenue Hospital are all private hospitals, Garissa County Referral Hospital, Kericho County Referral Hospital and Kenyatta University Teaching and Referral Hospital are government hospitals, while PCEA Chogoria and Tenwek Hospitals are faith-based organization. All the participating facilities provided either secondary or tertiary levels of care.

The study population was any general adult public visiting the outpatient clinics at the different healthcare facilities. The only eligibility criteria was adults ≥ 18 years of age and those who consented to be part of the study. Based on an assumption of 24.5% [8] and an attrition rate of 15%, the minimum sample size calculated was 328 participants. The participants were recruited based on convenience sampling.

The survey was in English and consisted of questions based on demographics and clinical characteristics, history of hypertension and current antihypertensive medication use. To reduce potential bias of self-reported data, confidentiality of participants and privacy of their responses was prioritized. Data were collected both through online and paper-based surveys. Each site had an investigator that was responsible for the administration of the survey. The site investigator, based on the resources available at the respective sites, decided to choose either using paper or online surveys. Each site investigator received preliminary training on the administration of the survey. The site investigator interviewed the patient and was responsible for filling in the survey. Online survey data were collected through the Research Electronic Data Capture (REDCap) platform (Vanderbilt and National Institute of Health) [13]. Online and written consent was obtained from all the participants. Approval for this study was obtained from the Institutional Scientific and Ethics Review Committee (ISERC) (Ref:2022/ISERC-81(v3)) at the Aga Khan University, Nairobi, Kenya's National Commission for Science, Technology and Innovation (NACOSTI) (Ref:217822) and all the hospital leaderships. Participants were allowed to withdraw from the study at any time without any consequences.

Sociodemographic factors included age, gender, race, education, marital status, income, residence, and body mass index. Behavioral risk factors included current daily smoker, current daily consumer of alcohol, adequate physical activity and insufficient fruit and vegetable intake. Adequate physical activity was defined as engaging in ≥ 3 days of vigorous activity of at least 20 minutes per day or ≥ 5 days of moderate intensity activity or walking of at least 30 minutes per day. Insufficient fruit and vegetable intake was defined as consuming < 5 servings of fruits and/or vegetables per day. Diabetes was reported as a prior diagnosis of diabetes by a health-care professional.

Blood pressures were measured, and three readings were taken on the left arm in a seated position, at 1 minute intervals. A large or extra-large cuff was primarily used based on the participant bicep diameter. All sites were encouraged to use either an OMRON basic BP machine or a Mindray BP machine based on the affordability of the specific clinic. An average of the three readings was taken as the final value for both systolic and diastolic blood pressures. Hypertension was defined by JNC-7 as mean systolic blood pressure (SBP) at least 140 mmHg, or diastolic blood pressure (DBP) at least 90 mmHg [14].

Continuous data were analyzed using medians and interquartile ranges (IQRs) whereas categorical data were analyzed as frequencies and percentages. The non-parametric Kruskal–Wallis test was used to compare the continuous variables and Fisher’s exact test was used to compare the categorical variables between group associations. Logistic regression was also employed to identify associations and odds ratio, and 95% CI was presented. Data analysis was performed using SPSS statistical software V. 20.0 (IBM, Armonk, NY, USA). The significance level was set at α = 0.05, and all tests were two-tailed.

**RESULTS**

*Demographic Characteristics*

A total of 1444 patients were recruited and included in the analysis. Table 1 presents the overall demographic characteristics of the participants. The median age of participants was 47.0 years (IQR: 34.0, 60.0) with 54.3% being females. More than half of the participants (75.1%) were married and about 70.6% had an education of secondary or higher. Most of the participants were Africans (96.4%), 65.4% were employed or self-employed and 54.8% were overweight or obese. About 42.9% reported living in the rural areas and the recruitment covered residence from 33 counties.

*Behavioral Characteristics and disease prevalence*

Table 2 summarizes the behavioral characteristics. About 6.4% reported being current daily smokers, whereas 8.1% reported being current daily alcohol users. Less than half, 44.4% reported having adequate physical activity and 48.7% reported having sufficient intake of fruits and vegetables. Based on comorbid conditions, 13.5% reported having diabetes, 12.8% reported having cardiovascular diseases, and 28.5% reported having a prior diagnosis of hypertension. 29.4% of patients had raised systolic blood pressures compared to 17.3% with increased diastolic pressures. Based on the average measures of systolic and diastolic blood pressures, the prevalence of hypertension was found in 29.4% of patients.

*Association between Hypertension with Demographic and Behavioral Characteristics*

Table 3 summarizes the association between hypertension with demographic and behavioral characteristics. Past history of hypertension was associated with hypertension diagnoses, where 48.5% diagnosed with hypertension did not have any past history as compared to those not diagnosed with hypertension, 81.1% did not have past history (p<0.01). Furthermore, of those who reported having a prior diagnosis, 53.1% had uncontrolled hypertension. Study sites were associated with hypertension, where 39.1% of participants with hypertension were from faith-based institutions, 31.5% from public institution and 29.4% from private institutions (p<0.001). Based on demographic characteristics, age, gender, education, marital status, body mass index and residence were associated with hypertension. The median age for those with hypertension was higher as compared to those with no hypertension (55.0 years vs 42.0 years; p<0.001). More males were reported to have hypertension as compared to not having hypertension (52.4% vs 42.9%; p=0.001). More patients that had hypertension reported having lower education (primary or less) as compared to those not having hypertension (37.3% vs 26.1%; p<0.001). More patients that had hypertension reported living in the rural areas as compared to those not having hypertension (51.5% vs 39.3%; p<0.001).

Based on behavioral characteristics, alcohol consumption, food intake, diabetes, cardiovascular disease, and prior diagnosis of hypertension were associated with hypertension. For participants diagnosed with hypertension, 10.6% reported using alcohol daily as compared to 7.1% of non-hypertensive participants reporting daily use of alcohol (p=0.025). More than half of the participants that did not have hypertension had sufficient intake of fruits and vegetables (52.5%) whereas only 39.5% of the hypertensive participants reported sufficient intake of fruits and vegetables (p<0.001).

**DISCUSSION**

*High prevalence of hypertension with low awareness*

Africa has the highest global prevalence of hypertension [15, 16]. An estimated 150 million adults in Africa are hypertensive [16], a figure predicted to rise to around 216.8 million by 2030 [17, 18]. Hypertension is a leading preventable risk factor for cardiovascular disease (CVD), which has a mortality rate of approximately 13.8% in Kenya (**Error! Reference source not found.**), higher than the global CVD mortality rate of 12.8% [19]. The current study found a hypertension prevalence of 29.4% similar to recent studies done in Kenya, finding a prevalence’s of 24.5% [8], 28.6% [7] and 30% [20]. Of note, mortality from non-communicable diseases (NCDs), including cardiovascular disease (CVD) as a major contributor, is projected to increase by more than 50% over the next decade. In Kenya, NCD’s make up 50% of hospitalisations and 55% of inpatient deaths [21] making it imperative to prioritise prevention and early intervention in order to decrease the disease burden of hypertension and resulting CVD’s.

Of the hypertensive patients in this study, almost half (48.5%) had a first-time diagnosis, , which was much higher than findings from similar studies in Kenya 15.6% (**Error! Reference source not found.**) to 29.6% [7]. Significant lack of awareness on hypertension has been implicated throughout LMICs, where it is estimated only 1 in 3 people are aware of their hypertension status [22]. The lack of awareness found in this study could be explained by a lack of screening, especially in the poorer populations of Kenya. Oyando and colleagues found that 73% of the poorest quantile of the population in Kenya had never been screened for hypertension, contrasting 38% of the richest quantile [21]. High expenditures involved in hypertension screening, including high transport costs due to physical inaccessibility of healthcare facilities, may prevent frequent health check-ups in poorer communities [23]. Additionally, the National Health Insurance Fund (NHIF) in Kenya does not cover hypertension testing making it an out-of-pocket expense and giving little incentive to have the test done. This can lead to a high prevalence yet low awareness, especially in rural Kenya where hypertension seems to be most predominant. It is important to increase hypertension screening in rural facilities, including governmental and Faith Based Hospitals (FBH’s). These FBH’s provide 30% to 70% of healthcare services in LMIC’s [24] and up to 40% in rural areas of Kenya [25]. The importance of such facilities is highlighted by the fact that the majority of hypertensive patients in this study (39.1%) had sought care at a faith-based hospital.

*Urban – rural differences in hypertensive prevalence*

Certainly, despite the majority of the participating patients being from urban areas, over half of the hypertensive group were from rural areas (42.9%). This reflects the decline in urban-rural difference in hypertension prevalence [7, 26], as well as the results of a recent meta-analysis showing higher increases in prevalence in the rural populations of the most developed LMIC’s [27]. Our study also showed that most hypertensive patient sought care in either FBHs or governmental healthcare facilities located in mostly rural areas in Kenya. One budding hypothesis for the higher prevalence in rural areas may be increased indoor air pollution from burning solid fuels in rural households. The incomplete combustion of fuels used for cooking and heating can lead to increased indoor particulate matter (PM) pollution which is positively correlated to the development of hypertension [28, 29]. A study done in rural and urban settlements in poor neighbourhoods in Kenya showed that most households live above the recommended World Health Organisation limit for indoor PM pollution [30]. Many rural homes in Kenya still rely on the burning of solid fuels such as wood [31], compared to cleaner or more energy-efficient fuels such as natural gas or electricity used in urban homes [32]. Air pollution from the burning of solid fuels is associated with increased risk of hypertension [33]. This underlines a need to increase awareness on the health effects of indoor air pollution from solid fuels especially in rural Kenya.

*Marital status and hypertension*

Our study showed a larger proportion of married participants, 82.5%, were hypertensive. These findings are similar to a Nigerian study showing an 88% increased likelihood of hypertension in both married men and women [34]. In contrast however, marriage has been purported to be associated with a better health status including lower cardiovascular risk factors [35]. Furthermore, marriage has been associated with lower night-time systolic blood pressure, a finding which was more significant in men [36]. The relationship between marital status and hypertension prevalence seemingly varies by gender and has been reported by other studies. Aziz and colleagues found that married men were less likely to have higher blood pressures [37]. Also, a twelve year follow up study in Iran found men who were never married had up to a 55% increased risk for hypertension compared to married men [38]. Meanwhile, Tuoyire and colleagues found that women who were married, cohabiting or previously married, had significantly higher chances of developing hypertension. The authors went on to conclude marital status as an independent risk factor for hypertension for women in Ghana [39]. Thus, these findings may suggest marriage has more beneficial effect on men’s health than women, and potentially even a disadvantageous effect on women’s health. Looking at our own results, however, there is a higher likelihood of hypertension in both men and women who are married, with a larger percentage (49.7%) of hypertensive married participants being male. Differing cultural and gender roles in marriage have been proposed as potential explanations for these differences in hypertension prevalence. These include: the increased pressure on men to provide for their families, and the increased pressure on women to care for children and spouses while also working. Alternatively, marriage may increase socio-economic status leading to more access to unhealthy processed foods that are associated with hypertension [34, 39]. Further investigation into these contrasting associations between marital status, hypertension prevalence and gender is necessary, while also considering the effects gender roles in marriage in different cultures.

*Age, gender, education, and hypertension*

Other demographic factors involved in the prevalence of hypertension were found to be age and gender. The risk of developing hypertension increases with age [40] and the patients with hypertension in this study had a higher median age (55 years) than those without hypertension (42 years), a comparable finding to other studies [7, 8, 26]. The growing aging population in Africa and Kenya predicts a worsening burden of hypertension and related CVD’s [41, 42]. That said, hypertension has also been recorded in younger age groups (18 – 35 years) [43], and 33% of primary school children in urban Kenya have been observed to have stage 1 hypertension (prehypertension) [44]. Healthcare facilities and future research should therefore not favour age as a main factor for the screening of hypertension. Furthermore, this study found a higher percentage of men with hypertension than women. This is a common finding [7, 16], some evidence even suggesting men twice more likely to have hypertension than women in Kenya [20]. It is suggested that women may get to know about their hypertension status earlier than men, possibly through antenatal screening or have better health seeking than men [7, 22]. There is also a proposed protective role of oestrogen in the pathophysiology of hypertension, that reduces after menopause [45].

Our study also finds significant associations between levels of education and prevalence of hypertension. Prevalence of hypertension increases with increasing education level, however, only up to secondary education, after which the trend reverses. The highest level of education in this study, tertiary, was associated with the lowest prevalence of hypertension. When comparing groups, a high percentage of our non-hypertensive participants had tertiary education (49.3%), and a high percentage of hypertensive patients (68.4%) had only secondary education and below. Findings are contentious, with some studies showing higher hypertension prevalences with higher education [7, 46, 47], while others show higher educational attainment linked to better health outcomes [48] including lower hypertension prevalence [49, 50]. In Kenya, households with heads who had higher levels of education were more likely to utilise healthcare services than those households whose heads had lower levels of education. Authors suggested that more educated household heads understood the importance of regular health checkups [51]. Additionally, conditions such as hypertension and its risk factors may be relatively unknown in people with little education [52] possibly leading to less knowledge of healthier lifestyles for prevention of hypertension, or the importance of early screening and intervention.

*Poor diet, lack of physical activity, obesity, and hypertension*

Over half of the patients in both the hypertensive and non-hypertensive groups reported not having adequate daily physical activity. Physical inactivity increases the risk of developing hypertension [53] and exercise can not only prevent or delay the onset of hypertension [54], but the reduction in blood pressure from increased physical activity can be as great as first-line antihypertensive medications [55]. The concerning lack of physical activity in this cohort extends to other studies done in Kenya, including one finding 80.3% of the population did not have sufficient physical activity [56], and another finding 86.8% of adolescents in the country are insufficiently active [18]. It is essential to educate the public on the health implications of physical inactivity, and perhaps place more importance on physical education in schools.

One significant difference in lifestyle between the groups in this study was diet. A lower percentage of the patients with hypertension had sufficient intake of fruit and vegetable (FV) compared to non-hypertensive patients. Consumption of more FV promotes a healthy diet and possibly leads to less consumption of unhealthy foods high in fat and salt. An inverse relationship between the consumption of FV and hypertension risk has been shown [57]. This is postulated to be because FV are high in fibre and essential vitamins and minerals such as potassium, magnesium, vitamin C and others [58, 59]. An increased intake of fibre has been shown to reduce blood pressure [59]. Studies have also shown an inverse relationship between the consumption of potassium and the prevalence of hypertension [60]. Diet is a crucial factor to consider in the study of hypertension prevalence, and added to this, is the consumption of alcohol. Moreover, moderate to heavy drinkers have a greater risk for hypertension [61], and not drinking alcohol was seen to lower the risk of hypertension in 18–35-year-old Kenyans by 70% [43]. In this study, a higher percentage of hypertensive patients were daily consumers of alcohol compared to non-hypertensive patients.

Supporting what our study showed, physical inactivity and poor diet are major contributors to overweight/obesity which has been known for a long time to be an independent risk factor for hypertension [1, 62]. In this study there was a higher percentage of obese patients in the hypertensive group. Higher BMI’s are associated with hypertension [7] and in LMIC’s the fastest increase in obesity comes from rural rather than urban areas [27]. The likelihood of an overweight/obese person having hypertension is 8 times compared to a healthy weight individual [26]. Additionally in the studied primary school children in Kenya, overweight children were seen to have a 1.83-fold greater risk for hypertension than healthy weight children [44]. The prevalence of obesity in Kenya was found at 31.13% [63], however with continuing urbanisation and shift from traditional to more westernised diets, this prevalence is likely to increase [64].

*Future considerations*

It is important to consider factors associated with hypertension prevalence in more detail in the Kenyan population. For example: though FV consumption is seen to be poor, another major diet driver for hypertension is sodium intake, as diets high in sodium are strongly related to hypertension [59]. Additionally, though this study measured daily consumption of alcohol, future research should also consider the units consumed per day in hypertensive and non-hypertensive individuals.

The PASCAR and WHF cardiovascular scorecard project found that 72.5% of people in Kenya live in rural areas [18]. Though it may be assumed, coming from rural areas does not necessitate very low income. Therefore, another avenue for research would be investigating the association between income bracket and hypertension prevalence in Kenya in rural and urban populations.

Following on, conventional lifestyle factors such as poor diet and physical inactivity, though contributing, may not fully explain the pathogenesis and prevalence of hypertension. Psychosocial stress has been found to increase the prevalence of hypertension and is also associated with poor hypertension control [65]. Therefore, examining subjective measures of psychosocial stress in future study participants alongside other behavioural and sociodemographic characteristics, would be valuable.

Furthermore, it would be interesting to investigate the differences in hypertension prevalence between people with higher education, that is secondary and above. Studies can attempt to replicate the finding of a reversed trend in hypertension prevalence between secondary and tertiary education and elucidate possible reasons for this. Additionally, as women have been found to have a closer association between educational level and hypertension, comparison between the genders would be useful [50]. Further gender comparison in hypertension prevalence in married people in Kenya would also be valuable for future comparative studies.

Lastly, exploration of the prevalence of prehypertension and its conversion into hypertension in the Kenyan population may inform more accurate intervention measures. As it stands, prevalence of prehypertension in Kenya is estimated at 54.5% [66] and is even seen in populations as young as primary school children [44]. Moreover, prehypertension is itself associated with increased risk for CVD’s [67]. Screening for prehypertension in a clinical setting therefore must also be encouraged to allow for earlier health interventions to prevent conversion to hypertension.

This study had several limitations. Firstly, the blood pressures were measured in one clinic setting and ideally would have been better to measure them in various or multiple clinic visits. Secondly, even though we have 8 participating sites, additional sites especially county hospitals, where most Kenyans seek medical attention, would have been more reflective of the burden of hypertension in Kenya.

**CONCLUSION**

This study found a high prevalence of hypertension in the population, especially in rural areas of Kenya, with low awareness. Factors associated with hypertension were higher age group, being male, being married, having secondary or lower education, insufficient consumption of FV, obesity and alcohol consumption. Primary hypertension is preventable and blood pressure tests are simple, quick, non-invasive and should be inexpensive. From this study and others done in Kenya, it is clear that public education strategies should be implemented to raise awareness on hypertension and its risk factors, with special focus on rural areas. Along with this, public health interventions to promote healthier lifestyles to all age groups in Kenya are vital in trying to control the rising burden of hypertension and related CVD’s. It is also crucial to increase hypertension screening in the country to allow for earlier intervention where possible. The fact remains that hypertension disease burden in Kenya will continue rising with increasing urbanisation and development of the country, its control must therefore be made a national priority.

**Contribution**

JS contributed to the development of the study, overall management of data collection, analysis of the data and was the overall project lead. SM, CS, JN, AO, LB, FA, HA, SJ, CM and RP were responsible of their respective site data management and collection. JS and CS contributed to the draft of the manuscript. All authors contributed to the manuscript and approved for submission.

**Declaration of interests**

The authors declare no competing interests.

**REFERENCES**

1.	World Health Organization. *Hypertension*. 2021 [cited 2022 11 May]; Available from: <https://www.who.int/news-room/fact-sheets/detail/hypertension>.

2.	Gatimu, S.M. and T.W. John, *Socioeconomic inequalities in hypertension in Kenya: a decomposition analysis of 2015 Kenya STEPwise survey on non-communicable diseases risk factors.* Int J Equity Health, 2020. **19**(1): p. 213.

3.	Dzudie, A., et al., *Roadmap to achieve 25% hypertension control in Africa by 2025.* Cardiovasc J Afr, 2017. **28**(4): p. 262-272.

4.	Statistics, B.O., *KENYA STEPwise SURVEY FOR NON COMMUNICABLE DISEASES RISK FACTORS 2015 REPORT.* 2015.

5.	Ogola, E., et al., *[OP. 3C. 03] Blood pressure screening results from Healthy Heart Africa: screening locations, participant characteristics, and hypertension classification in Kenya.* Journal of Hypertension, 2017. **35**: p. e30-e31.

6.	Hickey, M.D., et al., *Effect of a patient-centered hypertension delivery strategy on all-cause mortality: Secondary analysis of SEARCH, a community-randomized trial in rural Kenya and Uganda.* PLoS Med, 2021. **18**(9): p. e1003803.

7.	Pengpid, S. and K. Peltzer, *Prevalence, awareness, treatment and control of hypertension among adults in Kenya: cross-sectional national population-based survey.* East Mediterr Health J, 2020. **26**(8): p. 923-932.

8.	Mohamed, S.F., et al., *Prevalence, awareness, treatment and control of hypertension and their determinants: results from a national survey in Kenya.* BMC Public Health, 2018. **18**(Suppl 3): p. 1219.

9.	van de Vijver, S.J., et al., *Prevalence, awareness, treatment and control of hypertension among slum dwellers in Nairobi, Kenya.* J Hypertens, 2013. **31**(5): p. 1018-24.

10.	Okello, S., et al., *Hypertension prevalence, awareness, treatment, and control and predicted 10-year CVD risk: a cross-sectional study of seven communities in East and West Africa (SevenCEWA).* BMC Public Health, 2020. **20**(1): p. 1706.

11.	Guwatudde, D., et al., *The burden of hypertension in sub-Saharan Africa: a four-country cross sectional study.* BMC Public Health, 2015. **15**: p. 1211.

12.	Chowdhury, M.A.B., et al., *Urban rural differences in prevalence and risk factors of self-reported hypertension among Kenyan women: a population-based study.* J Hum Hypertens, 2021. **35**(10): p. 912-920.

13.	Harris, P.A., et al., *The REDCap consortium: Building an international community of software platform partners.* J Biomed Inform, 2019. **95**: p. 103208.

14.	Chobanian, A.V., et al., *Seventh report of the Joint National Committee on Prevention, Detection, Evaluation, and Treatment of High Blood Pressure.* Hypertension, 2003. **42**(6): p. 1206-52.

15.	Ferdinand, K.C., *Uncontrolled hypertension in sub-Saharan Africa: Now is the time to address a looming crisis.* J Clin Hypertens (Greenwich), 2020. **22**(11): p. 2111-2113.

16.	Parati, G., et al., *How to Improve Awareness, Treatment, and Control of Hypertension in Africa, and How to Reduce Its Consequences: A Call to Action From the World Hypertension League.* Hypertension, 2022. **79**(9): p. 1949-1961.

17.	AstraZeneca. *Healthy Heart Africa - Access to Healthcare* Available from: <https://www.astrazeneca.com/sustainability/access-to-healthcare/healthy-heart-africa.html>.

18.	Mbau, L., et al., *PASCAR and WHF Cardiovascular Diseases Scorecard project.* Cardiovasc J Afr, 2021. **32**(3): p. 161-167.

19.	World Health Organization, *Indicator Metadata Registry*

20.	Nshimirimana, A., et al., *Prevalence of Systemic Hypertension in Kenya: A Multicentre Analysis of the Karen Hospital Database.* Journal of Kenya Association of Physicians March, 2019. **2**(1): p. 23.

21.	Oyando, R., E. Barasa, and J.E. Ataguba, *Socioeconomic Inequity in the Screening and Treatment of Hypertension in Kenya: Evidence From a National Survey.* Front Health Serv, 2022. **2**: p. 786098.

22.	Schutte, A.E., et al., *Hypertension in Low- and Middle-Income Countries.* Circ Res, 2021. **128**(7): p. 808-826.

23.	Oyando, R., et al., *Evaluating the effectiveness of the National Health Insurance Fund in providing financial protection to households with hypertension and diabetes patients in Kenya.* Int J Equity Health, 2023. **22**(1): p. 107.

24.	Ndege, J.M., W. Tenambergen, and K. Njoroge, *The role of organizational culture in moderating corporate governance practice influence on faith-based hospitals performance in Kenya.* Open Access Library Journal, 2022. **9**(7): p. 1-17.

25.	Nicol, J.U., et al., *The impact of faith-based organizations on maternal and child health care outcomes in Africa: taking stock of research evidence.* Pan Afr Med J, 2022. **43**: p. 168.

26.	Bosu, W.K., et al., *Hypertension in older adults in Africa: A systematic review and meta-analysis.* PLoS One, 2019. **14**(4): p. e0214934.

27.	Ranzani, O.T., et al., *Urban-rural differences in hypertension prevalence in low-income and middle-income countries, 1990-2020: A systematic review and meta-analysis.* PLoS Med, 2022. **19**(8): p. e1004079.

28.	Wang, B., et al., *Indoor air pollution affects hypertension risk in rural women in Northern China by interfering with the uptake of metal elements: A preliminary cross-sectional study.* Environ Pollut, 2018. **240**: p. 267-272.

29.	Niu, Z., et al., *Association between long-term exposure to ambient particulate matter and blood pressure, hypertension: an updated systematic review and meta-analysis.* Int J Environ Health Res, 2023. **33**(3): p. 268-283.

30.	Mutahi, A.W., et al., *Indoor and outdoor air quality for sustainable life: a case study of rural and urban settlements in poor neighbourhoods in Kenya.* Sustainability, 2021. **13**(4): p. 2417.

31.	Dida, G.O., et al., *Factors predisposing women and children to indoor air pollution in rural villages, Western Kenya.* Arch Public Health, 2022. **80**(1): p. 46.

32.	Ali, M.U., et al., *Health impacts of indoor air pollution from household solid fuel on children and women.* J Hazard Mater, 2021. **416**: p. 126127.

33.	Li, L., et al., *Indoor air pollution from solid fuels and hypertension: A systematic review and meta-analysis.* Environ Pollut, 2020. **259**: p. 113914.

34.	Adeke, A.S., et al., *Socio-demographic and lifestyle factors associated with hypertension in Nigeria: results from a country-wide survey.* J Hum Hypertens, 2022.

35.	Manfredini, R., et al., *Marital Status, Cardiovascular Diseases, and Cardiovascular Risk Factors: A Review of the Evidence.* J Womens Health (Larchmt), 2017. **26**(6): p. 624-632.

36.	Causland, F.R., F.M. Sacks, and J.P. Forman, *Marital status, dipping and nocturnal blood pressure: results from the Dietary Approaches to Stop Hypertension trial.* J Hypertens, 2014. **32**(4): p. 756-61.

37.	Azizi, Z., et al., *The role of sex and gender in hypertension.* J Hum Hypertens, 2023. **37**(8): p. 589-595.

38.	Ramezankhani, A., F. Azizi, and F. Hadaegh, *Associations of marital status with diabetes, hypertension, cardiovascular disease and all-cause mortality: A long term follow-up study.* PLoS One, 2019. **14**(4): p. e0215593.

39.	Tuoyire, D.A. and H. Ayetey, *Gender differences in the association between marital status and hypertension in Ghana.* Journal of biosocial science, 2019. **51**(3): p. 313-334.

40.	National Institute of Aging. *High Blood Pressure and Older Adults*. 2022; Available from: <https://www.nia.nih.gov/health/high-blood-pressure/high-blood-pressure-and-older-adults#:~:text=The%20chance%20of%20having%20high>.

41.	Gyasi, R.M. and D.R. Phillips, *Aging and the Rising Burden of Noncommunicable Diseases in Sub-Saharan Africa and other Low- and Middle-Income Countries: A Call for Holistic Action.* Gerontologist, 2020. **60**(5): p. 806-811.

42.	Kiilu, C., *PRESENTATION ON DEMOGRAPHIC AGEING, THE SITUATION OF OLDER PERSONS AND RECENT POLICY DEVELOPMENT IN KENYA*. 2021.

43.	Ondimu, D.O., G.M. Kikuvi, and W.N. Otieno, *Risk factors for hypertension among young adults (18-35) years attending in Tenwek Mission Hospital, Bomet County, Kenya in 2018.* Pan Afr Med J, 2019. **33**: p. 210.

44.	Gewa, C.A., et al., *Patterns and predictors of elevated blood pressure and hypertension among primary school children in urban Kenya.* J Hypertens, 2022. **40**(12): p. 2513-2520.

45.	Di Giosia, P., et al., *Gender Differences in Epidemiology, Pathophysiology, and Treatment of Hypertension.* Curr Atheroscler Rep, 2018. **20**(3): p. 13.

46.	Nyarko, S.H., *Prevalence and Sociodemographic Determinants of Hypertension History among Women in Reproductive Age in Ghana.* Int J Hypertens, 2016. **2016**: p. 3292938.

47.	Laxmaiah, A., et al., *Socio-economic & demographic determinants of hypertension & knowledge, practices & risk behaviour of tribals in India.* Indian J Med Res, 2015. **141**(5): p. 697-708.

48.	Duarte, C.D., et al., *Lifecourse Educational Trajectories and Hypertension in Midlife: An Application of Sequence Analysis.* J Gerontol A Biol Sci Med Sci, 2022. **77**(2): p. 383-391.

49.	Nakagomi, A., et al., *Social determinants of hypertension in high-income countries: A narrative literature review and future directions.* Hypertens Res, 2022. **45**(10): p. 1575-1581.

50.	Kautzky-Willer, A., et al., *Women show a closer association between educational level and hypertension or diabetes mellitus than males: a secondary analysis from the Austrian HIS.* BMC Public Health, 2012. **12**: p. 392.

51.	Mwami, M.N. and M.O. Oleche, *Determinants of utilization of health Care Services in Kenya.* International Journal of Academic Research in Business and Social Sciences, 2017. **7**(10): p. 132-156.

52.	Tedesco, M.A., et al., *Educational level and hypertension: how socioeconomic differences condition health care.* J Hum Hypertens, 2001. **15**(10): p. 727-31.

53.	Oparil, S., et al., *Hypertension.* Nat Rev Dis Primers, 2018. **4**: p. 18014.

54.	Diaz, K.M. and D. Shimbo, *Physical activity and the prevention of hypertension.* Curr Hypertens Rep, 2013. **15**(6): p. 659-68.

55.	Pescatello, L.S., et al., *Exercise for Hypertension: A Prescription Update Integrating Existing Recommendations with Emerging Research.* Curr Hypertens Rep, 2015. **17**(11): p. 87.

56.	Wanjau, M.N., L.N. Aminde, and J.L. Veerman, *The avoidable disease burden associated with overweight and obesity in Kenya: A modelling study.* EClinicalMedicine, 2022. **50**: p. 101522.

57.	Li, B., et al., *Fruit and Vegetables Consumption and Risk of Hypertension: A Meta-Analysis.* J Clin Hypertens (Greenwich), 2016. **18**(5): p. 468-76.

58.	Zhao, D., et al., *Dietary factors associated with hypertension.* Nat Rev Cardiol, 2011. **8**(8): p. 456-65.

59.	Reynolds, A.N., et al., *Dietary fibre in hypertension and cardiovascular disease management: systematic review and meta-analyses.* BMC Med, 2022. **20**(1): p. 139.

60.	Burnier, M., *Should we eat more potassium to better control blood pressure in hypertension?* Nephrol Dial Transplant, 2019. **34**(2): p. 184-193.

61.	Puddey, I.B., et al., *Alcohol and Hypertension-New Insights and Lingering Controversies.* Curr Hypertens Rep, 2019. **21**(10): p. 79.

62.	Narkiewicz, K., *Obesity and hypertension--the issue is more complex than we thought.* Nephrol Dial Transplant, 2006. **21**(2): p. 264-7.

63.	Mkuu, R., et al., *Prevalence and factors associated with overweight and obesity in Kenya.* Prev Med Rep, 2021. **22**: p. 101340.

64.	Mbogori, T., et al., *Nutrition transition and double burden of malnutrition in Africa: A case study of four selected countries with different social economic development.* AIMS Public Health, 2020. **7**(3): p. 425-439.

65.	Marwaha, K., *Examining the Role of Psychosocial Stressors in Hypertension.* J Prev Med Public Health, 2022. **55**(6): p. 499-505.

66.	Mecha, J.O., et al., *Burden of prehypertension among adults in Kenya: a retrospective analysis of findings from the Healthy Heart Africa (HHA) Programme.* BMC Public Health, 2020. **20**(1): p. 281.

67.	Doumas, M., N. Katsiki, and D.P. Mikhailidis, *Prehypertension, the risk of hypertension and events.* Prehypertension and Cardiometabolic Syndrome, 2019: p. 37-55.

| Study Site | Private Facility | 557 | 38.6% |
| --- | --- | --- | --- |
|  | Public Facility | 160 | 31.2% |
|  | Faith Based Facility | 195 | 30.3% |
| Age (years) (n=1427) (median [IQR]) | | 47.0 [34.0, 60.0] | |
| Sex (n=1442) | Female | 783 | 54.3% |
|  | Male | 659 | 45.7% |
| Race (n=1441) | African | 1389 | 96.4% |
|  | Asian | 30 | 2.1% |
|  | Caucasian | 13 | 0.9% |
|  | Others (Arab/Somali) | 9 | 0.6% |
| Education (n=1423) | No School | 180 | 12.6% |
|  | Primary | 239 | 16.8% |
|  | Secondary | 377 | 26.5% |
|  | Tertiary | 627 | 44.1% |
| BMI (n=1413) | Underweight | 83 | 5.9% |
|  | Healthy | 555 | 39.3% |
|  | Overweight | 457 | 32.3% |
|  | Obese | 318 | 22.5% |
| Marital Status (n=1440) | Married | 1081 | 75.1% |
|  | Others | 92 | 6.4% |
|  | Single | 267 | 18.5% |
| Employment (n=1438) | Employed / Self Employed | 941 | 65.4% |
|  | Retired | 175 | 12.2% |
|  | Unemployed | 322 | 22.4% |
| Income Per Month (Kenya Shillings) (Gross Amount) | 0 - 15,000 | 395 | 27.4% |
|  | 100,000 - 200,000 | 115 | 8.0% |
|  | 16,000 - 50,000 | 235 | 16.3% |
|  | 51,000 - 100,000 | 202 | 14.0% |
|  | Above 200,000 | 44 | 3.0% |
|  | Prefer not to answer | 453 | 31.4% |
| Place of Residence (n=1435) | Rural | 616 | 42.9% |
|  | Urban | 819 | 57.1% |
| Regions (n=1428) | Central | 202 | 14.1% |
|  | Coast | 160 | 11.2% |
|  | Eastern | 232 | 16.2% |
|  | Nairobi | 269 | 18.8% |
|  | North Eastern | 182 | 12.8% |
|  | Nyanza | 33 | 2.3% |
|  | Rift Valley | 350 | 24.5% |

Table1: Overall demographic characteristics of 1444 patients.

| Current Daily Smoker | No | 1351 | 93.6% |
| --- | --- | --- | --- |
|  | Yes | 93 | 6.4% |
| Current Daily Consumer of Alcohol | No | 1327 | 91.9% |
|  | Yes | 117 | 8.1% |
| Adequate Physical Activity | No | 803 | 55.6% |
|  | Yes | 641 | 44.4% |
| Sufficient Fruits and Vegetable Intake | No | 741 | 51.3% |
|  | Yes | 703 | 48.7% |
| Diabetes | No | 988 | 68.4% |
|  | Yes | 195 | 13.5% |
|  | Unknown | 261 | 18.1% |
| Cardiovascular Disease | No | 936 | 64.8% |
|  | Yes | 185 | 12.8% |
|  | Unknown | 323 | 22.4% |
| Prior Diagnosis of Hypertension | No | 1032 | 71.5% |
|  | Yes | 412 | 28.5% |
| Current use of prescribed medication | No | 59 | 14.3% |
|  | Yes | 353 | 85.7% |
| Overall Hypertension | No | 1019 | 70.6% |
|  | Yes | 425 | 29.4% |

Table 2: Overall behavioral characteristics of the study cohort.

|  | | **Hypertension** | | | | **P Value** |
| --- | --- | --- | --- | --- | --- | --- |
|  |  | **NO** | | **YES** | |  |
|  |  | **(n=1019)** | | **(n=425)** | |  |
| Age (years) (n=1427) (median [IQR]) | | 42.0 [32.0, 57.0] | | 55.0 [43.0, 65.0] | | <0.001 |
| Gender (n=1442) | Female | 581 | 57.1% | 202 | 47.6% | 0.001 |
|  | Male | 437 | 42.9% | 222 | 52.4% |  |
| Race (n=1441) | African | 977 | 96.1% | 412 | 97.2% | 0.306 |
|  | Others | 40 | 3.8% | 12 | 2.8% |  |
| Education (n=1423) | No School | 123 | 12.2% | 57 | 13.6% | <0.001 |
|  | Primary | 140 | 13.9% | 99 | 23.7% |  |
|  | Secondary | 247 | 24.6% | 130 | 31.1% |  |
|  | Tertiary | 495 | 49.3% | 132 | 31.6% |  |
| Marital Status (n=1440) | Married | 731 | 71.9% | 350 | 82.5% | <0.001 |
|  | Others | 55 | 5.4% | 37 | 8.7% |  |
|  | Single | 230 | 22.6% | 37 | 8.7% |  |
| Employment (n=1438) | Employed / Self Employed | 673 | 66.2% | 268 | 63.5% | 0.052 |
|  | Retired | 110 | 10.8% | 65 | 15.4% |  |
|  | Unemployed | 233 | 22.9% | 89 | 21.1% |  |
| Residence Status (n=1435) | Rural | 397 | 39.3% | 219 | 51.5% | <0.001 |
|  | Urban | 613 | 60.7% | 206 | 48.5% |  |
| Current Daily Smoker | No | 961 | 94.3% | 390 | 91.8% | 0.073 |
|  | Yes | 58 | 5.7% | 35 | 8.2% |  |
| Current Daily Consumer of Alcohol | No | 947 | 92.9% | 380 | 89.4% | 0.025 |
|  | Yes | 72 | 7.1% | 45 | 10.6% |  |
| Adequate Physical Activity | No | 552 | 54.2% | 251 | 59.1% | 0.088 |
|  | Yes | 467 | 45.8% | 174 | 40.9% |  |
| Sufficient Fruits and Vegetable Intake | No | 484 | 47.5% | 257 | 60.5% | <0.001 |
|  | Yes | 535 | 52.5% | 168 | 39.5% |  |
| Diabetes | No | 718 | 70.5% | 270 | 63.5% | 0.007 |
|  | Yes | 181 | 17.8% | 80 | 18.8% |  |
|  | Unknown | 120 | 11.8% | 75 | 17.6% |  |
| Cardiovascular Disease | No | 713 | 70.0% | 223 | 52.5% | <0.001 |
|  | Yes | 220 | 21.6% | 103 | 24.2% |  |
|  | Unknown | 86 | 8.4% | 99 | 23.3% |  |
| Prior Diagnosis of Hypertension | No | 826 | 81.1% | 206 | 48.5% | <0.001 |
|  | Yes | 193 | 18.9% | 219 | 51.5% |  |
| BMI (n=1413) | Underweight | 73 | 7.3% | 10 | 2.4% | <0.001 |
|  | Healthy | 420 | 42.3% | 135 | 32.2% |  |
|  | Overweight | 320 | 32.2% | 137 | 32.7% |  |
|  | Obese | 181 | 18.2% | 137 | 32.7% |  |
| Study Site | Private | 432 | 42.4% | 125 | 29.4% | <0.001 |
|  | Public | 316 | 31.0% | 134 | 31.5% |  |
|  | Faith | 271 | 26.6% | 166 | 39.1% |  |

Table 3: Association between hypertension with demographic and behavioral characteristics
