# Supplementary material for: Prevalence and risk factors of hypertension among clients seeking care at Selected Healthcare Facilities in Kenya
Source: PLoS One. 2025 Oct 29;20(10):e0334255. doi: 10.1371/journal.pone.0334255 (PMC12571272; doi:10.1371/journal.pone.0334255)
Supplement: Attachment — Submitted filename: Hypertension PLOS ONE - Response Feedback 11th Aug.docx [file pone.0334255.s005.docx]

**PREVALENCE, DEMOGRAPHIC AND ASSOCIATIONS OF PATIENTS WITH HYPERTENSION SEEKING CARE AT DIFFERENT HEALTHCARE FACILITIES IN KENYA**

**[PONE-D-25-09467]**

We thank the editor and reviewer for pointing out the comments below and we have tried to address the comments and strengthen the manuscript.

**Response to Comments**

**Overall Editor Comments**

Journal Requirements:

When submitting your revision, we need you to address these additional requirements.

1. Please ensure that your manuscript meets PLOS ONE's style requirements, including those for file naming. The PLOS ONE style templates can be found at

<https://journals.plos.org/plosone/s/file?id=wjVg/PLOSOne_formatting_sample_main_body.pdf>

<https://journals.plos.org/plosone/s/file?id=ba62/PLOSOne_formatting_sample_title_authors_affiliations.pdf>

*Response: We have revised the manuscript to the journals style requirements. We have incorporated from both the templates.*

2. Please include captions for your Supporting Information files at the end of your manuscript, and update any in-text citations to match accordingly. Please see our Supporting Information guidelines for more information: <http://journals.plos.org/plosone/s/supporting-information>.

*Response: We have included the captions.*

3. We note that there is identifying data in the Supporting Information file <Hypertension Data.xls>. Due to the inclusion of these potentially identifying data, we have removed this file from your file inventory. Prior to sharing human research participant data, authors should consult with an ethics committee to ensure data are shared in accordance with participant consent and all applicable local laws. Data sharing should never compromise participant privacy. It is therefore not appropriate to publicly share personally identifiable data on human research participants. Please remove or anonymize all personal information, ensure that the data shared are in accordance with participant consent, and re-upload a fully anonymized data set. Please note that spreadsheet columns with personal information must be removed and not hidden as all hidden columns will appear in the published file.

*Response: We have revised and ensured the data file is completely anonymous.*

4. If the reviewer comments include a recommendation to cite specific previously published works, please review and evaluate these publications to determine whether they are relevant and should be cited. There is no requirement to cite these works unless the editor has indicated otherwise.

*Response: We appreciate the reviewer’s comments as they have helped us to strengthen our manuscript.*

Reviewer #1:

Introduction

1. Paragraphs 2 and 3 are a repetition of the same point. Could be combined in one paragraph or summarised.

*Response: We have revised the introduction for better flow.*

Methods

2. Clearly described. Methods are aligned with the study aim.

*Response: Thank you for the comment.*

Results

3. The planned sample size was 328 but 1444 participants were recruited. What was the rationale for increasing the sample size 4 times.

*Response: Based on an assumption of 24.5% and an attrition rate of 15%, the minimum sample size calculated was 328 participants. Since we were recruiting from three types of facilities (private, public and faith-based), the minimum sample required was 984 participants. We have revised this in the methods section.*

4. Significant findings (hypertension was associated with obesity and age) were not stated in the Results section. Please provide narrative describing the data in the tables.

*Response: This has been revised in the results section*

5. The abbreviation FV is not widely used. Consider writing in full.

*Response: We have revised and written it in full.*

Conclusion

6. The conclusions answer the research question.

*Response: Thank you for the comment.*

References

7. Please format the references according to the journal guidelines and include DOIs.

*Response: We have revised the references accordingly.*

If reviewer comments were submitted as an attachment file, they will be attached to this email and accessible via the submission site

*Response: Furthermore, the reviewer had suggested few changes and comments to the manuscript. Below are the comments and our responses.*

1. The title is misleading. The sample are not people living with HPT, but clients attending the OPD. Change to “Prevalence and risk factors of hypertension among clients seeking care at selected facilities in Kenya”

*Response: We have revised the Title.*

Abstract Comments:

2. Abstract: Include a section about how the data was analysed.

*Response: We have included a summary of the analysis in the Methods of the abstract.*

3. Is goal blood pressure determined by the clients or medical doctors? Are you referring to hypertension control as directed by the WHO.

*Response: We have included in the abstract the use of guidelines defined by the Joint National Committee.*

4. This statement is misleading. The facility is faith-based not all the clients are pastors. Kindly rewrite for clarity.

*Response: We have clarified the sentence.*

Introduction Comments:

5. The switch from global, to regional and national level evidence from the literature is inconsistent. You have Kenya in the previous sections, Nigeria in this section and from line 89 you have global evidence. Use the funnel

*Response: We have reworked the entire Introduction to flow it better.*

6. I disagree with this assertion; there are countless studies on hypertension in SSA. Look at the RODAM project and EDULINK project

*Response: We have omitted the sentence.*

Methods Comments:

7. Long confusing sentence. Group them by location first; for example Nairobi. And only indicate the number that are private, public or faith-based.

*Response: We have revised and grouped them based on the type of healthcare facility.*

8. Include references for the behavioral risk factors.

*Response: We have included the references.*

9. Kindly describe how BMI was measured in the methods. BMI is a lifestyle or behavioural risk but not demographic characteristics

*Response: We have described in the methods and put BMI with the other behavioral characteristics.*

10. Start sentence with words.

*Response: This has been revised.*

11. Restructure the Results for Table 3.

*Response: We have revised the results.*

12. This statement is incorrect. Past history is hyp diagnosis.

*Response: We have revised the sentences for better clarity.*

Discussion

13. Kindly present your logistic regression before the discussion. Although you have presented the discussion in sections; it is best to present a key section where you summarise your results before providing the interpretation

*Response: We have not done logistic regression for these analyses. We have excluded it from the methods. This was an oversight. We have tried to rework on the discussion for better structure.*

References:

14. Most of the references are incomplete. Add the url link for the reports. For example, how can we access ref 4.

*Responses: We have tried to incorporate and correct the references.*

15. What is the heading for this table

*Response: We have included the headings.*

16. Why do the sample size vary across variables. This is very confusing. Keep only the same sample size throughout the study.

*Response: Since participation in the survey was voluntary, some questions were left unanswered, resulting in missing responses for certain variables. To ensure transparency, we have reported the sample size (n) for each variable, allowing readers to see where data are missing.*

17. This difference between 52 and 49 is quite small. This association will not be retained in a regression model. Kindly provide evidence of this in a regression model

*Response: The observed difference is not between 51.5% and 48.5%, but rather between 51.5% of participants with elevated blood pressure who were from rural areas and 39.3% of participants without elevated blood pressure who were from rural areas. This represents a 12.2 percentage point difference.*
