# Supplementary material for: Prevalence and risk factors of hypertension among clients seeking care at Selected Healthcare Facilities in Kenya
Source: PLoS One. 2025 Oct 29;20(10):e0334255. doi: 10.1371/journal.pone.0334255 (PMC12571272; doi:10.1371/journal.pone.0334255)
Supplement: Attachment — Submitted filename: Hypertension PLOS ONE - Response Feedback 15th Sept.docx [file pone.0334255.s006.docx]

**PREVALENCE, DEMOGRAPHIC AND ASSOCIATIONS OF PATIENTS WITH HYPERTENSION SEEKING CARE AT DIFFERENT HEALTHCARE FACILITIES IN KENYA**

**[PONE-D-25-09467]**

We thank the editor and reviewer for pointing out the comments below and we have tried to address the comments and strengthen the manuscript.

**Response to Comments**

Dear Authors,

Kindly revise the manuscript based on the comments in the attached file.

In the response to reviewer's file do only indicate "this correction has been made", copy and paste the revision. Your document should be such that decisions can be made based on it.

1. Revision from patients to clients

*Response. This correction has been made.*

2. Kindly consider how this is phrased. In rural areas, faith-based facilities are the dominant care providers. If you flip this around, htn is increasing in rural areas, which is what your findings indicate. This was not the case in the past and it would be great if you could highlight that

*Response: We have rephrased and added “ In rural areas, faith-based facilities are the dominant care providers”*

3. Hospital?

*Response: The statement is correct. For each hospital, we have provided the location (city or town) where it is situated, for example, Aga Khan University Hospital, Nairobi.*

4. Did you consider those with previous diagnosis?

*Response: We considered both with and without previous diagnosis. We have revised in the statement where it was highlighted.*

5. Kindly present the logistic regression results.

*Response: We have added the Odds Ratio (OR) in Table 3 and revised the results section accordingly.*

6. The data does not support this conclusion: only 72 participants were diagnosed by the study who had no previous diagnosis

*Response: From the 1444 clients, 425 (29.4%) met the criteria for hypertension at clinic visit. Among the 425 clients that met the criteria for hypertension, 219 (51.5%) had a prior diagnosis of hypertension whereas 206 (48.5%) did not have a prior diagnosis. We have included the numbers in the paragraph, and this reflects in the table.*

7. Another motivation could also be that these facilities provide relatively inexpensive services

*Response: The correction has been made and have added this in the discussion.*

8. Kindly indicate whether these are not the only hospitals in the counties. These centres may have their own pull factors, such as short waiting times compared to crowded public hospitals. Going to seek care at any facility is not a risk factor of hypertension

*Response: We agree that the site of care is not a risk factor for hypertension. Our intention was to highlight the role of faith-based hospitals as major providers of care in rural Kenya, which may explain why the largest proportion of participants were recruited from these facilities. We have revised the text to clarify that these hospitals are not the only health facilities in their counties.*

9. This was not part of your results

*Response: We agree that the statement about prehypertension in primary school children was not part of our study results but was included as supporting evidence from previous literature. To avoid confusion, we have revised the text to clearly separate our findings from published data as this is presented in future considerations.*

10. Which government unit should be doing this? Is blood pressure assessment part of regular OPD services? If yes, that’s facility level screening. Now find out how community level screening can be done and suggest that

*Response: We have corrected and revised the paragraph.*
